# The Immunodiagnostic Utility of Antinuclear Antibody Patterns: A Prediction for Renal Involvement in Systemic Lupus Erythematosus Patients in the Western Region of Saudi Arabia

**DOI:** 10.7759/cureus.43532

**Published:** 2023-08-15

**Authors:** Jamil Al-Mughales

**Affiliations:** 1 Department of Clinical Microbiology and Immunology, King Abdulaziz University Faculty of Medicine, Jeddah, SAU; 2 Department of Clinical Laboratories, Diagnostic Immunology Division, King Abdulaziz University Hospital, Jeddah, SAU

**Keywords:** pattern, staining, antinuclear antibodies, immune markers, nephritis, renal involvement, systemic lupus erythematosus

## Abstract

Objectives

Previous studies have noted associations between the immunofluorescence patterns of antinuclear autoantibodies (ANA) and the autoimmune responses seen in systemic lupus erythematosus (SLE). In this study, the authors tested the hypothesis of whether ANA patterns predict renal involvement in SLE patients.

Method

A retrospective study was carried out on consecutive SLE patients who had ANA staining pattern data and who were screened for renal involvement defined as all-stage proteinuria or chronic kidney disease (CKD) at a referral tertiary center in western Saudi Arabia from December 2021 to February 2022. Demographic data and levels of lupus immune markers including ANA titers, anti-double-stranded deoxyribonucleic acid antibodies (anti-dsDNA), complements C3 and C4, anticardiolipin (aCL) immunoglobulin (Ig) G and IgM, anti-β2 glycoprotein (β2-IgM and β2-IgG), and lupus anticoagulant (LA) antibodies were collected.

Result

Among 243 patients included, 25.1% had renal involvement (95% confidence interval {CI}=19.8-31.0). A mixed ANA pattern was associated with a higher prevalence of renal involvement (46.2%), followed by homogenous (26.5%) and speckled (25.6%) patterns, compared with 4.5% for the other patterns (p=0.044). No further association of renal involvement was observed with other biological markers. Adjusted logistic regression showed age (odds ratio {OR}=0.95; 95% CI=0.92-0.97) and mixed ANA pattern (OR=26.66; 95% CI=2.53-281.11) to be independently associated with renal involvement, explaining 12.6% of the variance.

Conclusion

A mixed homogenous/speckled ANA staining pattern is associated with an increased risk of renal involvement, independent of ANA titer or other lupus immune markers. The potential clinical applications of the ANA staining pattern in SLE should be explored in various subtypes of SLE and patient groups.

## Introduction

Systemic lupus erythematosus (SLE) is a complex autoimmune disorder with multiorgan involvement, resulting from the interplay of genetic susceptibility, environmental factors, and immune/hormonal triggers [1,2]. It imposes significant health and economic challenges, including increased morbidity, cardiovascular complications, and reduced health-related quality of life (HRQoL), with variable epidemiological figures across ethnicities [3-10]. Recent research has deepened our understanding of SLE's immunopathogenesis, highlighting an aberrant immune response against nuclear autoantigens and the production of antinuclear autoantibodies (ANA). This involves disruptions in myeloid and lymphoid immunity, increased activation of autoreactive T and B lymphocytes, impaired clearance of immune complexes, and enhanced interferon pathways, all culminating in systemic autoimmunity [2,11,12]. Accordingly, SLE management focuses on mitigating disease activity, preventing organ damage, and enhancing survival and HRQoL [2].

Renal involvement, or lupus nephritis, represents a common and severe complication of SLE, observed between 7% and 50% of patients, depending on the stage and duration of the disease, as well as the ethnic group [13,14]. The prevalence of biopsy-proven lupus nephritis was estimated to be between 20% and 40% [15,16]. Lupus nephritis may be present at the time of SLE diagnosis or develop a few months to several years after diagnosis [17]. It may occur in flares with no clinical renal manifestation [18] as it may manifest as overt chronic kidney disease (CKD), the risk of which is potentiated by other factors such as a history of cardiovascular disease, atherosclerosis, or sepsis [14,19]. Therefore, the early diagnosis and timely management of lupus nephritis are paramount, since a delayed diagnosis increases the risk of end-stage renal disease (ESRD) [20]. An international meta-analysis study that included 187 articles and 18,300 patients showed that lupus nephritis was associated with a five-year risk of ESRD of about 10%-12% [21].

Several immunopathological mechanisms are intricately involved in lupus nephritis. These include systemic autoantibodies targeting different kidney structures, leading to the persistent presence of nuclear material in the extracellular space, which triggers further autoantigen-specific adaptive immune response locally and leads to the fixation of immune memory. Renal injury results from the local deposition of the immune complex and the activation of the complement, which leads to intrarenal inflammation [22]. The recent understanding of the correlations between immunological and histological disorders enabled defining several markers to enhance the early diagnosis of renal involvement in SLE. Thus, the occurrence of lupus nephritis is associated with higher autoimmune profile, indicated by higher anti-double-stranded deoxyribonucleic acid antibodies (anti-dsDNA) and greater consumption of complement indicated by low C3 [18,23]. Another marker of renal involvement is anti-C1q antibodies, which are more accurate in ruling out the diagnosis with a negative predictive value of 100%, besides being strong positive markers for lupus nephritis flares and proteinuria [24-26].

While monitoring these markers may enhance early diagnosis, certain genotypes have been identified to increase the risk or severity of lupus nephritis [27]. However, due to the impracticality of genotyping studies in routine practice, it remains of interest to prospectively characterize the patients' profiles associated with greater risk for developing lupus nephritis. This would enable a better appraisal of the renal prognosis and adaptation or renal monitoring in high-risk patients. In a previous work, the author evidenced a correlation of autoimmune activity in SLE patients with the immunofluorescence pattern of ANA [28]. This study tests the hypothesis of whether ANA patterns were also predictive for renal involvement in SLE patients.

## Materials and methods

Design and participants

This retrospective study was carried out at the Diagnostic Immunology Division, Department of Laboratory Medicine, King Abdulaziz University Hospital (KAUH), Jeddah, Saudi Arabia, from December 2021 to February 2022. KAUH is a referral immunodiagnostic center in the western region of Saudi Arabia. The study protocol was reviewed and ethically approved by the institutional review board of KAUH (reference number: 130-21).

Participants

Consecutive patients who were diagnosed with SLE and treated in KAUH from January 2018 to December 2020 and who had sufficient data to test the primary objective of the study were included. Hence, patients who had no results for ANA pattern or renal involvement were not included. SLE diagnosis and classification were carried out according to the 2019 European League Against Rheumatism/American College of Rheumatology (EULAR/ACR) criteria [29]. A non-probability, convenience sampling was used to include all eligible patients.

Data collection

An Excel sheet (Microsoft® Corp., Redmond, WA) was designed to collect the following data: 1) patient's demographic data (age, gender, nationality, and ethnic group); 2) SLE-specific biological markers, including ANA titers and staining patterns, anti-dsDNA, complement levels (C3 and C4), anticardiolipin (aCL) immunoglobulin (Ig) G and IgM, anti-β2 glycoprotein (β2-IgM and β2-IgG), and lupus anticoagulant (LA) antibodies; 3) nonspecific biological markers including C-reactive protein (CRP) level, absolute leucocyte count, hemoglobin level, prothrombin time (PT), and partial thromboplastin time (PTT); and 4) clinical data regarding renal status including creatinine level and estimated glomerular filtration rate (eGFR).

Diagnostic immunology assays

All immunodiagnostic analyses were carried out at the same laboratory and using the same methods for all patients and in compliance with the respective manufacturers' guidelines. ANA test was performed by indirect immunofluorescence (IIF) technique utilizing human epithelial cells (human epithelial type 2 {HEp-2}) fixed on glass slides using Aesku kits (Aesku Diagnostics, Windlesham, Germany). ANA patterns were categorized into peripheral, speckled, homogenous, nucleolar, and centromere.

Anti-dsDNA were analyzed by the enzyme-linked immunosorbent assay (ELISA) technique, using the INOVA System Quanta Lite™ Ds-DNA Kit (San Diego, CA).

β2-IgG and β2-IgM were analyzed using Alegria instrument (Ogentec, Mainz, Germany), using the ELISA technique. C3 and C4 levels were analyzed using BNII nephelometry instrument (Siemens, Berlin, Germany); LA was analyzed using the commercial insert (Diagnostica Stago, Asnières-sur-Seine, France).

In addition, CRP level, leucocyte count, hemoglobin level, PT, and PTT were analyzed in accordance with the standard laboratory methods (Diagnostica Stago, Asnières-sur-Seine, France).

Variables

Dependent Variable

The primary outcome was renal involvement, indicated by the presence at diagnosis or occurrence during the follow-up period of proteinuria of >500 mg/24 hours, [30] or the diagnosis of a chronic kidney disease (CKD), defined as chronic reduction of eGFR of <60 mL/minute/1.73 m^2^ [14].

Independent Variables

Independent variables included ANA pattern and titer, demographic data, and other specific and nonspecific biological markers.

Statistical methods

The Excel database was checked for critical data missing, duplicates, and outliers and then transferred to Statistical Package for Social Sciences (SPSS) version 21 (IBM SPSS Statistics, Armonk, NY) for Windows for data analysis. Descriptive statistics were carried out to present frequencies and percentages for categorical variables and mean and standard deviation (SD) and/or median and interquartile range (IQR) for numerical variables. The association of renal involvement with categorical variables was analyzed using chi-square or Fisher's exact test, as applicable. The association of renal involvement with numerical variables used independent t-test or Mann-Whitney test, as applicable. A p-value of <0.05 was considered statistically significant.

## Results

Participants' characteristics

The study included 243 patients, with a mean (SD) age of 39.36 (15.72) years, and 86.4% were female. The majority were from an Arabian ethnic group (69.5%) (Table 1).

**Table 1:** Participants' demographic characteristics (N=243) SD: standard deviation

Parameter/category	Frequency	Percentage	Mean (range)	SD
Demographic data				
Age (years)			39.36 (8-87)	15.72
Gender				
Male	33	13.6		
Female	210	86.4		
Nationality				
Saudi	133	54.7		
Yemeni	36	14.8		
Chadian	10	4.1		
Pakistani	10	4.1		
Sudanese	10	4.1		
Indian	6	2.5		
Palestinian	8	3.3		
Others	30	12.3		
Ethnic group				
Arabian tribes	169	69.5		
Middle Eastern	18	7.4		
Afro-Arab	19	7.8		
African	12	4.9		
South Asian	20	8.2		
Southeast Asian	5	2.1		

Specific and nonspecific biological markers

The biological profiles of the patients are presented in Table 2. These showed percentages of patients who had high levels of CRP (33.2%), prothrombin time (38.5%), partial prothrombin time (PTT, 20.6%), and lupus anticoagulant (22.1%), in addition to positive rheumatoid factor (14.6%), aCL IgG (8.7%), aCL IgM (2.3%), β2-IgG (positive or strongly positive, 9.7%), and β2-IgM (positive or strongly positive, 5.3%). The median (IQR) anti-dsDNA, C3, and C4 were 423.40 (421.13) IU/mL, 0.97 (0.43) mg/dL, and 0.19 (0.14) mg/dL, respectively.

**Table 2:** Participants' specific and nonspecific biological markers (N=243) dsDNA, double-stranded deoxyribonucleic acid antibodies; aCL, anticardiolipin; IgG, immunoglobulin G; IgM, immunoglobulin M; CRP, C-reactive protein; SD, standard deviation; IQR, interquartile range; PTT, partial thromboplastin time

Marker/level	N	Frequency	Percentage	Mean	SD	Median	IQR
Nonspecific markers							
CRP	232			17.34	37.23	4.57	10.07
Negative (<10)		155	66.8				
Positive (10+)		77	33.2				
Moderate (10 to <20)		43	18.5				
Frank (20 to <100)		23	9.9				
High (100+)		11	4.7				
Not done	11						
Prothrombin time (seconds)	179						
Low (<9.4)		1	0.6				
Normal (9.4-12.5)		109	60.9				
High (>12.5)		69	38.5				
Not done	64						
PTT (seconds)	180						
Low (<25)		15	8.3				
Normal (25-37)		128	71.1				
High (>37)		37	20.6				
Not done	63						
Neutrophils	235			4.13	4.52	3.18	2.80
Lymphocytes	233			1.82	0.89	1.68	1.02
Hemoglobin (g/dL)	236			11.51	1.88	11.70	2.40
Creatinine level	232			92.33	144.04	58.35	22.8
Specific markers							
Anti-dsDNA	242			524.23	296.78	423.40	421.13
C3	237			2.14	11.15	0.97	0.43
C4	237			0.22	0.26	0.19	0.14
Rheumatoid factor	137			44.63	232.00	11.00	1.40
Negative (<15 IU/mL)		117	85.4				
Positive (>15 IU/mL)		20	14.6				
aCL IgG	172						
Negative (<15 IU/mL)		144	83.7				
Weak positive (15-40 IU/mL)		13	7.6				
Positive (>40 IU/mL)		15	8.7				
aCL IgM	176						
Negative (<15 IU/mL)		160	90.9				
Weak positive (15-40 IU/mL)		12	6.8				
Positive (>40 IU/mL)		4	2.3				
β2-IgG	124						
Negative (<15 U/mL)		97	78.2				
Weak positive (15 to <40 U/mL)		15	12.1				
Positive (40 to <80 U/mL)		4	3.2				
Strongly positive (80+ U/mL)		8	6.5				
β2-IgM	131						
Negative (<15 U/mL)		118	90.1				
Weak positive (15 to <40 U/mL)		6	4.6				
Positive (40 to <80 U/mL)		5	3.8				
Strongly positive (80+ U/mL)		2	1.5				
Lupus anticoagulant	163						
Normal (0-45)		127	77.9				
High (>45)		36	22.1				

Renal involvement and its association with ANA titers and patters

Of the total patients, 25.1% had renal involvement (95% CI=19.8-31.0). While no significant association was observed between renal involvement and ANA titer (p=0.374), mixed pattern was associated with higher prevalence of renal involvement (46.2%), followed by homogenous (26.5%) and speckled (25.6%) patterns, while the "other" patterns had low prevalence of renal involvement (4.5%), and the result was statistically significant (p=0.044) (Table 3).

**Table 3:** Titers and patterns of antinuclear antibodies in SLE patients (N=243) *Statistically significant result (p<0.05) SLE: systemic lupus erythematosus

Parameter	Total		No renal involvement (N=182, 74.9%)		Renal involvement (N=61, 25.1%)		p-value
	N	%	N	%	N	%	
Titer							
1:40	1	0.4	1	100.0	0	0.0	
1:80	1	0.4	1	100.0	0	0.0	
1:160	20	8.2	16	80.0	4	20.0	
1:320	39	16.0	34	87.2	5	12.8	
1:640	46	18.9	32	69.6	14	30.4	
1:1280	136	56.0	98	72.1	38	27.9	0.374
Pattern							
Speckled	125	51.4	93	74.4	32	25.6	
Homogenous	83	34.2	61	73.5	22	26.5	
Mixed pattern	13	5.3	7	53.8	6	46.2	
Nucleolar	13	5.3	13	100.0	0	0.0	
Centromere	2	0.8	2	100.0	0	0.0	
Peripheral	7	2.9	6	85.7	1	14.3	0.125
Pattern (four categories)							
Speckled	125	51.4	93	74.4	32	25.6	
Homogenous	83	34.2	61	73.5	22	26.5	
Mixed pattern	13	5.3	7	53.8	6	46.2	
Others	22	9.1	21	95.5	1	4.5	0.044*

Association of renal involvement with demographic data

Patients with renal involvement were significantly younger (mean {SD} age=31.70 {11.05} years) than those without renal involvement (41.93 {16.23} years), and the result was statistically significant (p<0.001). Furthermore, renal involvement was relatively more frequent among males compared to females (36.4% versus 23.3%) and in patients from South Asian and Southeast Asian ethnic groups compared with the other ethnic groups (40.0% each versus 0%-33.3%); however, these results were not statistically significant (p>0.05) (Table 4).

**Table 4:** Demographic factors associated with renal involvement (N=243) *Statistically significant result (p<0.05)

Parameter/category	No renal involvement		Renal involvement		p-value
	N	%	N	%	
Age (years)	41.93	16.23	31.70	11.05	<0.001*
Gender					
Male	21	63.6	12	36.4	
Female	161	76.7	49	23.3	0.109
Ethnic group					
Arabian tribes	127	75.1	42	24.9	
Middle Eastern	18	100.0	0	0.0	
Afro-Arab	14	73.7	5	26.3	
African	8	66.7	4	33.3	
South Asian	12	60.0	8	40.0	
Southeast Asian	3	60.0	2	40.0	0.093

Association of biological markers with renal involvement

Patients with renal involvement had more frequently positive CRP (40.7% versus 30.6%); however, this was not statistically significant (p=0.157). No further association of renal involvement was observed with other specific and nonspecific biological markers (Table 5).

**Table 5:** Association of biological markers with renal involvement ^F^Fisher's exact test; ^M^Mann-Whitney U test; *statistically significant result (p<0.05) aCL, anticardiolipin; IQR, interquartile range; PTT, partial thromboplastin time; CRP, C-reactive protein; dsDNA, double-stranded deoxyribonucleic acid antibodies; SD, standard deviation; IgG, immunoglobulin G; IgM, immunoglobulin M

Parameter	No renal involvement		Renal involvement		p-value
	Frequency	Percentage	Frequency	Percentage	
CRP					
Negative	120	69.4	35	59.3	
Positive	53	30.6	24	40.7	0.157
Anti-dsDNA					
Mean, SD	518.06	293.84	542.95	307.29	
Median, IQR	414.35	400.45	482.70	647.48	0.662^M^
Rheumatoid factor					
Negative	95	85.6	22	84.6	
Positive	16	14.4	4	15.4	1.000^F^
C3					
Mean, SD	1.66	9.28	3.52	15.32	
Median, IQR	0.99	0.45	0.89	0.30	0.138^M^
C4					
Mean, SD	0.22	0.30	0.19	0.10	
Median, IQR	0.20	0.14	0.18	0.10	0.436^M^
aCL IgG					
Negative	103	93.1	41	85.4	
Weak positive	12	9.7	1	2.1	
Positive	9	7.3	6	12.5	0.153
aCL IgM					
Negative	117	91.4	43	89.6	
Weak positive	8	6.3	4	8.3	
Positive	3	2.3	1	2.1	0.885
β2-IgG					
Negative	67	77.9	30	78.9	
Positive	19	22.1	8	21.1	0.897
β2-IgM					
Negative	67	77.9	30	78.9	
Positive	19	22.1	8	21.1	0.897
Prothrombin time					
Low	1	0.8	0	0.0	
Normal	72	59.0	37	64.9	
High	49	40.2	20	35.1	0.622
PTT					
Low	10	8.1	5	8.8	
Normal	86	69.9	42	73.7	
High	27	22.0	10	17.5	0.793
Lupus anticoagulant					
Normal (0-45)	88	75.9	39	83.0	
High (>45)	28	24.1	8	17.0	0.321

Independent factors of renal involvement

Adjusted logistic regression showed both age (OR=0.95; 95% CI=0.92-0.97) and mixed ANA pattern (OR=26.66; 95% CI=2.53-281.11) to be independently associated with renal involvement, explaining 12.6% of the variance (Table 6).

**Table 6:** Independent factors of renal involvement (adjusted binary logistic regression) Reference: category used as a reference to calculate OR; *statistically significant result (p<0.05) OR, odds ratio; CI, confidence interval; ANA, antinuclear autoantibody

Predictor	Level	OR	95% CI		p-value	Model goodness-of-fit R^2^
Age	Years	0.95	0.92	0.97	<0.001*	0.126
ANA pattern	Others	Reference	-	-	0.038*	
	Speckled	6.72	0.84	53.47	0.072	
	Homogenous	6.77	0.83	55.14	0.074	
	Mixed	26.66	2.53	281.11	0.006*	

## Discussion

Context and summary

Renal involvement in SLE and the consequent CKD and ESRD are associated with impaired HRQoL, high morbidity, and up to 26-fold risk of mortality compared to the general population, which entails a substantial health and economic burden [4,31]. This study retrospectively analyzed the association of renal involvement with ANA pattern and titer, in addition to other lupus markers. The findings support that mixed ANA pattern is independently associated with a 27-fold risk of renal involvement, independent of ANA titers and the levels of other lupus markers. Additionally, the risk of lupus nephritis decreases by 5% with every year of the patient's age. Furthermore, male patients and those from African or Asian ethnic groups had relatively greater risk; however, this was not statistically significant.

Prevalence of renal involvement in SLE patients

The prevalence of renal involvement was approximately 25%, which is consistent with data from the literature. Various case definitions and estimation methods for renal involvement are reported. By considering the time of SLE onset, the prevalence of renal symptoms varies between 25% and 50%, whereas the life incidences of lupus nephritis in adult SLE patients may reach 60% [22]. By considering case definition, cases diagnosed with biopsy were reported at a comparable prevalence of 20%-40% [15,16]. Kidney biopsy represents the golden standard in the diagnosis of lupus nephritis. It enables excluding differentials, appraising renal damage, and classifying the level of severity of nephritis [32]. The International Society of Nephrology/Renal Pathology Society (ISN/RPS) criteria and the National Institutes of Health (NIH) activity and chronicity indices are based on the histological features found in kidney biopsy [33]. Additionally, a repeat kidney biopsy is recommended to monitor the treatment efficacy, in combination with clinical and biological signs including serum creatinine concentration, proteinuria, or eGFR [34,35]. In this retrospective study, renal involvement was doomed based on clinical and biological data, and no biopsy results were available for the patients.

Renal involvement and associated demographic factors

The authors observed that patients with renal involvement were 10 years younger, on average with reference to their counterparts; every additional year of the patient's age is associated with 5% decrease in the risk of renal involvement. This may be due to younger patients having more likely a juvenile-onset SLE (JO-SLE). Data from studies comparing adults with JO-SLE showed that lupus nephritis is significantly more prevalent in juvenile forms (up to 82%) compared with adult-onset forms (up to 53%) [16,36,37]. The younger age of SLE patients who develop lupus nephritis represents an additional factor in the health and economic burden of the disease.

Regarding gender, males were likely at higher risk, although the results were not statistically significant. The literature reports frequent male preponderance of lupus nephritis, with a greater renal damage and a higher prevalence ranging from 27% to 75%, compared with 16% to 52% in females [38-40].

Another observation is the relatively high prevalence of renal involvement among South Asian and Southeast Asian ethnic groups (40.0%), followed by African descents (33.3%), although the differences were not statistically significant (p=0.093). The differential risk of lupus nephritis across ethnic groups stands for the genetic backgrounds of SLE. A multiethnic study by Lanata et al., involving 1,244 SLE patients, found that the prevalence of lupus nephritis was highest among Asian patients (62%), followed by African-Americans (55.2%) and Hispanics (52.1%), while it was lowest among Europeans (≤40%) [41]. This is in line with the findings in this study, which showed Asians and Africans having the highest risks. The same study by Lanata et al. carried out further gene-based analyses and showed several genotypes to be associated with lupus nephritis in certain ethnic groups [41]. Comparable observations have been reported in a cohort of US patients with Medicaid coverage (2000-2004), which showed a significantly greater risk for lupus nephritis among Hispanic, African-American, and Asian patients, and this risk is associated with a younger age at diagnosis [42]. This is also in line with an international cohort study by Hanly et al., showing African, Asian, and Hispanic ethnic groups to be at a greater risk for having lupus nephritis at inception (≤15 months of SLE diagnosis) [16].

Renal involvement and ANA pattern

The findings from this study demonstrate that mixed ANA pattern is independently associated with a 27-fold risk of lupus nephritis with reference to ANA patterns other than speckled and homogenous. Although the regression model, which also included age variable, explained only 12.6% of the outcome, such a finding indicates the potential utility of ANA immunofluorescence staining pattern in predicting the renal prognosis. Studies that analyzed the association of renal involvement with ANA pattern are scarce. A Swedish study by Frodlund et al. found that homogenous pattern was significantly associated with proliferative lupus nephritis, follow by speckled pattern, whereas mixed homogenous/speckled pattern was more frequently observed in low-class lupus nephritis [43]. This observation is not in contradiction with the findings from this study, as the severity of renal involvement was not assessed in this study. Furthermore, both homogenous and speckled patterns were observed to have relatively high risk of lupus nephritis, although this was not statistically significant. Conversely, Novianti et al. observed no significant correlation between ANA pattern and renal involvement, as indicated by proteinuria, in a group of 89 newly diagnosed, pediatric SLE patients [44]. Another intriguing case of a 34-year-old male was reported in Japan. This person had SLE cutaneous vasculitis with a mixed speckled/homogenous ANA pattern that progressed into lupus nephritis with the conversion of ANA pattern into discrete speckled type [45]. These observations indicate the potential interest of ANA pattern in predicting the organ damage in SLE.

In a previous study, the author demonstrated that ANA pattern had a significant immunodiagnostic value for disease activity as it showed several correlations with different SLE immune markers. Among these observations, mixed pattern was associated with remarkably higher levels of lupus anticoagulant [28]. This is consistent with a case-control study by Alba et al., which showed that lupus nephritis was associated with positive lupus anticoagulant with an odds ratio of 1.85 and 1.98 in unadjusted and adjusted analysis, respectively. Lupus nephritis was further associated with anti-dsDNA (OR=2.35) and anti-Smith autoantibody (OR=3.3) [46]. Another study by Farrugia et al. compared SLE patients with positive and negative lupus anticoagulants and showed that lupus anticoagulant was associated with a significant threefold risk of thrombosis and nonstatistically significant increase in serum creatinine levels. Histological examination showed no difference between the two groups in terms of activity or chronicity index [47]. Furthermore, lupus anticoagulant was demonstrated to be associated with adverse outcome in pregnant females with SLE, including lupus nephritis, preeclampsia, and proteinuria [22].

Other data showed that renal involvement was associated with other immune markers, notably low levels of C3 and C4, indicating high complement consumption and deposition [48]. Additionally, fluctuations in C3 and C4 levels were also demonstrated to be predictive for lupus nephritis [49]. The present study failed to demonstrate the correlation of renal involvement with any of the investigated immune markers.

Limitations

This study bears certain intrinsic limitations that merit acknowledgment. Primarily, given our reliance on retrospective data from the hospital's electronic databases, we faced challenges in procuring comprehensive clinical details. Specifically, the Systemic Lupus Erythematosus Disease Activity Index (SLEDAI) score and certain treatment data, which might offer crucial insights into the relation between renal involvement and ANA patterns in SLE patients, were not uniformly available for all participants. This limitation could potentially affect the depth of our findings. Additionally, the single-center nature of the study imposes constraints on the generalizability of our results. The observations derived from our cohort, based in one institution, might not be representative of SLE patients in other clinical settings or among different ethnic groups. Variations in patient demographics, clinical management, and disease manifestations could influence renal complications and ANA patterns in SLE. Therefore, caution should be exercised when extrapolating our findings to broader populations. Multicenter investigations with diverse patient samples are advocated to derive a more holistic understanding of the association between renal involvement and ANA patterns in SLE.

## Conclusions

The staining pattern of ANA in immunofluorescence assays provides valuable indications on the severity and prognosis of SLE and organ damage. A mixed homogenous/speckled staining pattern is associated with a substantial risk of renal involvement, independent of ANA titer or other lupus immune markers. Other patterns that are likely to be associated with renal involvement include speckled and homogenous. Furthermore, younger patients are at higher risk of renal nephritis and should benefit from stringent renal monitoring. The potential clinical applications of ANA staining patterns in SLE should be explored in various subtypes of SLE and ethnic groups, considering the multifaceted nature of the disease and the multiple prognostic factors.
